# Radiomics in Hypopharyngeal Cancer Management: A State-of-the-Art Review †

**DOI:** 10.3390/biomedicines11030805

**Published:** 2023-03-06

**Authors:** Carlos M. Chiesa-Estomba, Miguel Mayo-Yanez, Orlando Guntinas-Lichius, Vincent Vander-Poorten, Robert P. Takes, Remco de Bree, Gyorgy B. Halmos, Nabil F. Saba, Sandra Nuyts, Alfio Ferlito

**Affiliations:** 1 Otorhinolaryngology-Head & Neck Surgery Department, Hospital Universitario Donostia, Biodonostia Research Institute, Faculty of Medicine, Deusto University, 20014 San Sebastian, Spain; 2 Otorhinolaryngology-Head and Neck Surgery Department, Complexo Hospitalario Universitario A Coruña (CHUAC), 15006 A Coruña, Spain; 3 Department of Otorhinolaryngology, Jena University Hospital, 07743 Jena, Germany; 4 Section Head and Neck Oncology, Department of Oncology, KU Leuven—University of Leuven, 3000 Leuven, Belgium; 5 Department of Otolaryngology/Head and Neck Surgery, Radboud University Medical Center, 6525 GA Nijmegen, The Netherlands; 6 Department of Head and Neck Surgical Oncology, University Medical Center Utrecht, 3584 CX Utrecht, The Netherlands; 7 Department of Otorhinolaryngology/Head and Neck Surgery, University Medical Center Groningen, University of Groningen, 9712 CP Groningen, The Netherlands; 8 Department of Hematology and Medical Oncology, The Winship Cancer Institute, Emory University, Atlanta, GA 30322, USA; 9 Department of Radiation Oncology, University Hospitals Leuven, KU Leuven—University of Leuven, 3000 Leuven, Belgium; 10 Coordinator of the International Head and Neck Scientific Group, 35125 Padua, Italy

**Keywords:** head, neck, hypopharynx, radiomics, treatment

## Abstract

(1) Background: Hypopharyngeal squamous cell carcinomas usually present with locally advanced disease and a correspondingly poor prognosis. Currently, efforts are being made to improve tumor characterization and provide insightful information for outcome prediction. Radiomics is an emerging area of study that involves the conversion of medical images into mineable data; these data are then used to extract quantitative features based on shape, intensity, texture, and other parameters; (2) Methods: A systematic review of the peer-reviewed literature was conducted; (3) Results: A total of 437 manuscripts were identified. Fifteen manuscripts met the inclusion criteria. The main targets described were the evaluation of textural features to determine tumor-programmed death-ligand 1 expression; a surrogate for microvessel density and heterogeneity of perfusion; patient stratification into groups at high and low risk of progression; prediction of early recurrence, 1-year locoregional failure and survival outcome, including progression-free survival and overall survival, in patients with locally advanced HPSCC; thyroid cartilage invasion, early disease progression, recurrence, induction chemotherapy response, treatment response, and prognosis; and (4) Conclusions: our findings suggest that radiomics represents a potentially useful tool in the diagnostic workup as well as during the treatment and follow-up of patients with HPSCC. Large prospective studies are essential to validate this technology in these patients.

## 1. Introduction

Head and neck (H&N) cancers are the seventh most common type of malignancy worldwide [1]. Hypopharyngeal squamous cell carcinomas (HPSCC) account for approximately 3% to 5% of all H&N cancers [2,3], with a higher incidence in China and Eastern Europe, due to dietary habits, and higher tobacco and alcohol consumption [4]. Patients with HPSCC usually present with loco-regionally advanced disease and a correspondingly poor prognosis, despite recent diagnostic and therapeutic advances [5,6,7].

The increased application of organ preservation protocols, including induction chemotherapy (IC) followed by definitive radiotherapy (RT) or primary concurrent chemoradiotherapy (CRT), has been associated with a 5-year overall survival (OS) in well-selected patients from 38% to 51.9% [3,8]. The fact that survival outcome is comparable to that with traditional techniques, such as total laryngectomy with partial or total pharyngectomy and postoperative radiation [9], has led to a shift towards the use of organ preservation protocols, aiming to decrease functional impairment associated with surgery [3,4,5,6,7,8]. On the other hand, recurrence rates in loco-regionally advanced HPSCC after organ-preserving treatment remain high, and follow-up strategies vary between hospitals.

Currently, efforts are being made to improve tumor characterization and provide insightful information for outcome prediction based on biomarkers measured in clinical settings and technological advances in imaging that are routinely acquired as part of the diagnostic work-up for initial staging and in the follow-up process of H&N cancer patients. In this context, it is relevant to consider the use of radiomics, an emerging area of research involving the conversion of medical images into extracted data on shape, intensity, texture, and other parameters, to assess the distribution of voxel intensities, spatial information, and conduct a comparison between pixels in a region of interest (ROI) [10]. Radiomics-based approaches have the potential to allow clinicians to perform a holistic and non-invasive assessment of tumors, which could provide additional information to improve treatment and follow-up [11].

Before this approach is accepted as part of the routine clinical armamentarium, particularly for patients with such a poor prognosis as those with HPSCC, available data must be evaluated. This study aims to review the state-of-the art regarding the available literature on radiomic analysis in HPSCC.

## 2. Materials and Methods

**Figure 1:**
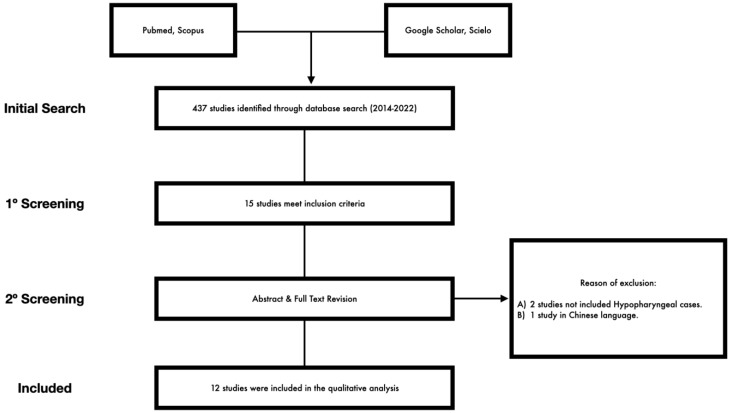
Prisma flowchart.

A systematic review of the peer-reviewed literature was conducted using keywords and following the recommendations of the Preferred Reporting Items for Systematic Reviews and Meta-Analyses statement (Figure 1) [12]. The selection criteria were based on the population, intervention, comparison, outcome, and time frame (PICOT) format [13]. This review was registered as a PROSPERO protocol (ID: 403002). The heterogeneity among studies, mainly attributable to a lack of randomization, limited our ability to statistically combine data into a formal meta-analysis. 

### 2.1. Population and Selection Criteria

Studies (both randomized or non-randomized clinical trials and prospective or retrospective cohort studies) on the use of radiomics in patients with HPSCC to estimate prognosis, outcome, or disease stage, or to analyze biological features were included. Publications were excluded if they were duplicates or studies on non-H&N or non-HPSCC cancer radiomics; animal studies; case reports, reviews, short communications, or letters; or gray literature. The different types of radiomic features described in each paper were summarized.

### 2.2. Intervention and Comparison

The intervention of interest was the predictive value of radiomics in HPSCC management. 

### 2.3. Outcomes

The primary outcome measures were the efficacy of radiomics as a surrogate, non-invasive method for predicting survival outcomes before and after treatment, estimating disease stage, or analyzing biological features (e.g., aggressive growth, perineural growth, and extracapsular extension) in HPSCC patients. 

### 2.4. Timing

The minimum median follow-up time considered to evaluate survival outcomes was 12 months after treatment.

### 2.5. Search Strategy

This review involved a systematic search of the electronic databases: MEDLINE via PubMed and via Ovid, Google Scholar, and Embase. We included articles published in English between January 2014 and August 2022. The search strategy was based on a combination of medical subject heading (MeSH) terms and other controlled vocabulary: “hypopharyngeal cancer” OR “head and neck cancer” OR “hypopharyngeal tumor” OR “radiomics”. Titles and abstracts were independently screened by two investigators (CMCE and MMY) to exclude publications that were irrelevant or duplicates. Information extracted from each study included the main target, imaging acquisition method, radiomic feature extraction/signature building/segmentation method, and treatment strategy. 

### 2.6. Assessment of Quality and Risk of Bias

Two authors (CMCE and MMY) evaluated the methodological quality of studies identified using the Oxford Centre for Evidence-Based Medicine Levels of Evidence [14]. The risk of bias was assessed by assigning a score using the Methodological Index for Non-Randomized Studies (MINORS) [15], an extensively validated instrument for literature assessment. For non-comparative studies, 8 domains were assessed, scoring the items as 0 (not reported), 1 (reported but inadequate), or 2 (reported and adequate). The optimal score for non-comparative studies is therefore 16. For the purposes of this review, a value of 10 or below was considered to represent a high risk of bias.

## 3. Results

A total of 437 manuscripts were identified and screened. Fifteen manuscripts met the inclusion criteria. Of these, two studies were excluded because they did not include H&N patients, and one because it was written in Chinese (Figure 1).

Twelve studies, all of them retrospective, were subjected to qualitative analysis [16,17,18,19,20,21,22,23,24,25,26,27]. The demographic characteristics of patients from these studies are summarized in Table 1. There was a high variation in the number of HPSCC patients included in each study, ranging from 2 to 167. Excluding one study that was based on the same sample as another study, we included a total of 889 patients who underwent treatment for HPSCC. Considering the 5 studies that provided data on age, the median age was 51 years old [23,24,25,26,27]. A total of 8 studies described patients by gender, yielding 645 (94.6%) males and 37 (5.4%) females [16,18,19,20,23,24,26,27]. The imaging acquisition method was CT in five studies [18,19,21,24,26], PET/CT in three [16,22,27], and MRI in two [20,25], while dual-energy CT [17] and quantitative ultrasound (US) [23] were each considered in one study. The treatment strategies used in the included studies were surgery, IC in combination with other treatment modalities, and CRT. 

The main targets described in the included studies were the evaluation of textural features as supplementary information to determine tumor-programmed death-ligand 1 (PD-L1) expression [16]; the role of radiomic features as a surrogate for microvessel density and heterogeneity of perfusion [17]; radiomic features leading to patient stratification into groups at high and low risk of progression [19,25]; radiomics predicting early recurrence [18] and 1-year locoregional failure and survival outcome, including progression-free survival (PFS) and overall survival (OS) in patients with locally advanced HPSCC [20]; and thyroid cartilage invasion [21], early disease progression [22], recurrence [23], induction chemotherapy response [26], treatment response [24], and prognosis [27]. The radiomic feature extraction, signature building, and segmentation methods are also described in Table 1. 

Looking more in depth regarding potential applications explored in recent years, Liu et al. highlight the ability of radiomic analysis to predict response to IC in HPSCC and hypothesized that radiomic signatures could also be capable of predicting PFS. Moreover, these authors found that all patients can be divided into high- and low-risk groups according to the optimal cutoff value of a proposed radiomics signature (called radscore) [26]. 

Hsu et al. reported an MRI-derived radiomic signature-based prediction model for the evaluation of 1-year loco-regional control and survival in patients with HPSCC who received organ preservation therapy as a first-line treatment, and they established a tumor volume cut-off value of >35 cm^3^ as a predictor of poor prognosis [20]. Moreover, the authors highlight the use of radiomic analysis and functional MRI modalities, such as diffusion-weighted imaging, and their potential to provide additional information about the prognostic imaging phenotype in patients with advanced HPSCC [19], as Leithner et al. [28] and Dulhanty et al. [29] demonstrated in breast and prostate cancers, respectively. In the same vein, Chen et al. developed an MRI-based radiomic nomogram to predict overall survival in HPSCC. They demonstrated better predictive capability by implementing a nomogram that included clinical and radiomic variables rather than the use of either separately [25].

Guo et al. sought to demonstrate the capacity of CT-radiomic features in the prediction of thyroid cartilage invasion in laryngeal squamous carcinoma and HPSCC. They based their hypothesis on the low sensitivity of CT to determine thyroid cartilage invasion (49–71%), usually due to differing rates of ossification of the cartilage, and found higher gray-level non-uniformity values in patients with thyroid cartilage invasion [21]. In a different setting, Bahig et al. suggest the potential role of dual-energy CT, a technology that allows tissue characterization through material decomposition and voxel-by-voxel quantification of iodine concentration, and tumor vessel characterization in conjunction with a radiomic-based approach before RT to predict loco-regional recurrence rates in laryngeal squamous carcinoma and HPSCC [16]. Similarly, Mo et al. demonstrated the potential role of CT radiomics to predict the risk of progression in HPSCC treated with organ preservation therapy [19]. 

In another study, Zhong et al. demonstrated the good predictive value of PET/CT combined with radiomics and machine learning analysis to predict the early progression (1 year) in patients treated with organ preservation therapy for HPSCC [21]. Further, Chen et al. showed the value of PET/CT radiomics to determine PD-L1 expression in this subset of patients [16]. Finally, Fatima et al. reported a clinical trial to explore the feasibility of US-delta or quantitative US radiomics during the treatment with RT to predict recurrence in H&N cancer patients. Despite just two patients with HPSCC having been included in that study, the potential value of US-based radiomics should be mentioned in this analysis [23]. 

According to the Oxford Centre for Evidence-Based Medicine system, seven studies were considered to provide level three evidence and five were considered to provide level four evidence. The MINORS scores indicating the risk of bias are listed in Table 2. 

## 4. Discussion

According to our systematic review, radiomics has the potential to support HPSCC management. Properly used, this technology has the promising potential to evaluate the characteristics of the tumor and its surrounding microenvironment, stratify patients by risk of progression, make predictions about treatment response, and improve survival estimates [16,27].

Nowadays, the UICC/AJCC TNM classification and staging system continues to be the first choice for clinical risk assessment and survival counseling [30]. Nonetheless, patients with HPSCC within the same UICC stage group have variable treatment prognoses, [30,31,32] highlighting the need for novel predictive assessment methods to improve patient stratification and propose individualized treatment plans. For this purpose, the existing clinical datasets from multiple imaging modalities routinely performed in HPSCC patients can be used in an exploratory radiomic analysis to support clinical decision-making by multidisciplinary teams.

The suffix “omics” is used to describe various sources of large volumes of data, being added to a name that characterizes the original materials for data collection. In line with this, the “radiomics” field finds its way into clinical research when large amounts of data from radiological imaging (intensity, shape, and texture) become available for analysis [33]. Specifically, texture analysis represents a set of tools to improve the characterization of tumor heterogeneity and consists in extracting texture indices from imaging modalities such as CT, MRI, and PET/CT [34]. Results from this type of analysis can be used as a surrogate biomarker to complement, facilitate, and accelerate advances towards precision medicine for cancer due to the non-invasive nature of this approach [10,35]. 

To translate this concept into medical or clinical research based on radiomics, the process needs to be broken down into five steps: (1) data collection; (2) ROI segmentation: delineation of the target area in the images; (3) feature selection: high-throughput extraction of lesion features; (4) feature reduction: selection of features with high reliability from the feature set for model training to improve the generalization ability of the model; and (5) establishment of a definitive model [36,37]. All these steps make it mandatory to define quality management protocols for radiomics application in H&N cancer patients, while also defining the time required per patient/exploration and the potential automatization of some parts of the process. 

Moreover, there are several different useful features. First-order features describe the pattern of distribution of gray-level pixel values within a whole tumor. The maximum and kurtosis values describe the highest intensities of the whole tumor, which are related to the hemodynamic parameters of the tumor and are relevant to the prediction of the efficacy of chemotherapy [38]. The pretreatment gray-level co-occurrence matrix (glcm) informational measure of correlation 1 (Imc1) has been studied to characterize tumor aggressiveness and predict induction chemotherapy response. Higher values of this feature are possibly associated with a good response [10]. In contrast, tumors with a higher value of neighborhood gray-tone difference matrix (ngtdm) business might have a poor response to neoadjuvant chemoradiation [39]. The gray-level non-uniformity feature has been studied to predict prognosis in patients with HPSCC, and in these cases, a lower gray-level uniformity indicates a poorer prognosis [19,40]. Similarly, lower values of glcm maximum correlation coefficient (MCC) may indicate tumors with more aggressive behavior, which can predict local failure after radiotherapy in locally advanced H&N cancers [41]. 

Regarding the potential information that we can obtain from the radiomics analysis about tumor growth behavior, potential treatment outcome, or the analysis of the surrounding microenvironment, we can highlight some radiomics features such as *the maximum and kurtosis values* useful to describe the highest intensities of the whole tumor, which are related to the hemodynamic parameters of the tumor, allowing us to predict chemotherapy efficacy [26]. The pretreatment *gray-level co-occurrence matrix (glcm)_Imc1*, a feature that allows us to characterize the tumor aggressiveness, also helps us to predict chemotherapeutic response, with a higher value associated with good treatment response [27]. We can also use the *gray-tone-difference matrix (ngtdm)_busyness*, a feature that can help us to predict neoadjuvant chemotherapy response, with this treatment being especially useful for patients with a negative response to treatment [28]. The *gray-level non-uniformity* feature, which is useful for prognosis prediction, has a low uniformity among gray levels and is a predictor of worse prognosis [20,29]. Finally, the *glcm_MCC* is a useful feature to estimate local failure after radiation therapy treatment in H&N cancer patients [30]. 

Summarizing the evidence available, radiomics is a non-invasive and cost-effective technique that has great potential to expand the scope of medical imaging in HPSCC patients [35]. Its application can provide important day-to-day information regarding rapid anatomic change and tumor response during treatment. Nonetheless, some issues need to be addressed. 

Radiomic features require further research into the correlation between genetic and biological imaging data. The clinical value of this tool in terms of reproducibility and variability across different settings in HPSCC needs to be established in prospective studies. Further, there is an ongoing debate about which imaging tool offers the best performance for radiomic analysis: CT, MRI, or PET/CT [42]. Nowadays, we know that contrast-enhanced CT images could provide more clear contours of tumors and important information associated with treatment response and prognosis than non-contrast CT images [40,43]. In this vein, imaging protocols and analysis must be standardized in order to be able to use the same radiomics models across different centers worldwide. 

Functional organ preservation represents a relevant target of IC. In HPSCC, the larynx preservation rate is an important outcome to be measured. However, there is a lack of information in almost all the studies included; therefore, it is necessary to investigate this in future research studies. We also have evidence about the possibility to combine radiomics signatures and clinical data using clinical nomograms to improve our results [24,26]. 

Due to the heterogeneity of HNSCC, there is a need for collection and organization of data from samples collected at multiples hospitals. In relation to this, an international European consortium designed the BD2Decide European multicenter project to explore the potential of big data in HNSCC for clinical outcome estimation and research. The aim of this project is to summarize the characteristics of a unique large international multi-omics database for the analysis of treatment differences in a selected series of patients with locally advanced cancer treated with curative intent. In this setting, radiomics is a promising image data mining method for the prediction of therapeutic response and prognosis, and its use in HPSCC should be included in this kind of project [44]. 

## 5. Conclusions

Based on the limited reports in the literature, our findings suggest that radiomics represents a potentially useful tool in the diagnostic workup as well as during the treatment and follow-up of patients with HPSCC. Large prospective studies are essential to validate this technology in these patients.

## Figures and Tables

**Table 1:** Studies investigating the role of radiomics in hypopharyngeal cancer. Abbreviations: Not described = ND; metabolic tumor volume = MTV; gray-level co-occurrence matrix = GLCM; gray-level run-length matrix = GLRLM; gray-level size zone matrix = GLSZM; and LASSO = least absolute shrinkage and selection operator. Computed tomography = CT; magnetic resonance imaging = MRI, positron emission tomography = PET-CT; ultrasound = US; RT =radiotherapy; CRT = chemoradiotherapy; LR = linear regression; OS = overall survival; PFS = progression-free survival; and LFS = laryngectomy-free survival.

Study	Main Objective	Number of Patients	Gender (M/F)	Age	Imaging Acquisition Method	Radiomic Feature Extraction/Signature Building/Segmentation Method	Treatment Strategy	Conclusion
Chen et al. [16] Taiwan—2017 retrospective study	Associations of tumor PD-1 ligands, IHC studies, and textural features in 18F-FDG PET	23	M = 23	ND	PET/CT	MTV; the heterogeneity of a tumor was evaluated using its textural features; GLCM, GLRLM, and GLSZM.	RT or CRT	p16 and Ki-67 staining percentages detected using IHC and several 18F-FDG PET/CT-derived textural features were explored. The PD-L1 expressions were positively correlated with p16 and Ki-67, whereas the textural index of correlation was a negative predictor for PD-L1 expression of ≥5%.
Bahig et al. [17] Canada—2018 retrospective study	Prediction of loco-regional recurrence	5	ND	ND	Dual energy—CT scan	Kurtosis, GTVp, and GTVn; iodine concentration (in mg/mL) extracted from GTVp and GTVn structures by determining the iodine partial electron density from each voxel, using a two-material decomposition method.	RT	Radiomics can represent a potential surrogate of microvessel density and heterogeneity of perfusion evaluation method for outcome prediction.
Li et al. [18] China—2020 retrospective study	Preoperative prediction of early recurrence	167	160/7	ND	CT	LASSO, LR; manual VOI delineation.	Surgery	Authors identified the noninvasive, predictive role of CT-based radiomics in the preoperative prediction of early recurrence of patients
Mo et al. [19] China—2020 retrospective study	Prediction of progression-free survival	113	106/7	ND	CT	Features extracted corresponds to texture, intensity, shape, and wavelet; LASSO. Wavelet HHH_glszm_GrayLevelNonUniformityNormalized Wavelet-LLH_firstorder_Maximum Wavelet-HLL_firstorder_Median Wavelet-HLH_glszm_LargeAreaEmphasis	CRT	According to the authors, the radiomic model showed good performance in stratifying patients into high- and low-risk groups of progression in hypopharyngeal cancer patients treated with chemoradiotherapy.
Hsu et al. [20] Taiwan—2020 retrospective study	Prediction of locoregional failure	116	111/5	ND	MRI	LASSO, skewness, and kurtosis.	CRT	The authors established and validated that a non-invasive RS model provides a novel and convenient approach to predict 1-year LRF and the survival outcome, including the PFS, LFS, and OS, in patients with locally advanced HPSCC who received organ preservation treatment.
Guo et al. [21] China—2020 retrospective study	Prediction of thyroid cartilage invasion	26	ND	ND	CT	LASSO GLCM, GLRLM, and GLSZM.	Preoperative diagnosis.	Models based on CT radiomic features were able to improve the accuracy of predicting thyroid cartilage invasion from LHSCC.
Zhong et al. [22] U.K.—2020 retrospective study	Prediction of early disease progression	40	ND	ND	PET-CT	PET parameters selected by the ML model were metabolic tumor volume (MTV); conventional minimum standardized uptake value (SUVmin); gray-level zone length matrix (GLZLM); small-zone low gray-level emphasis (SZLGE); histogram kurtosis; and histogram energy. CT parameters selected by the ML model were maximum CT attenuation value; GLZLM small-zone emphasis (SZE); mean CT attenuation value; GLZLM SZLGE; and GLZLM gray-level non-uniformity (GLNU). Clinical parameters selected by the ML model were duration of radiation treatment, nodal (N) stage, smoking, age, and sex. The parameters included in the combined model were MTV, maximum CT value, SUVmin, GLZLM SZLGE, and histogram kurtosis.	CRT	FDG PET-CT determined that radiomic features are potential predictors of early disease progression in patients with locally advanced larynx and hypopharynx SCC.
Fatima et al. [23] Canada—2021 retrospective study	Recurrence prediction	2	48/3	61	Quantitative US	In the quantitative ultrasound spectroscopy a total of seven spectral parameters were calculated within each ROI window. These include spectral slope (SS); spectral intercept (SI) at 0 MHz; mid-band fit (MBF); average acoustic concentration (AAC); average scatterer diameter (ASD); attenuation coefficient estimate (ACE); and spacing among scatterers (SAS). Texture analysis: GLCM, energy, homogeneity, and contrast.	RT	Machine learning classifiers trained with QUS spectral and texture parameters were shown to predict recurrence for patients with HNSCC receiving RT with an accuracy of 82% at week 4 of treatment.
Liu et al. [24] China—2022 retrospective study	Pretreatment predictor of progression-free survival in locally advanced hypopharyngeal carcinoma	112	103/9	61	CT	LASSO, Wavelet-LLH_glszm_ GrayLevelNonUniformityNormalized Gradient_glcm_Imc1 Wavelet-LLL_ngtdm_Busyness Wavelet-LHL_firstorder_Maximum Wavelet-LHL_firstorder_Kurtosis Wavelet-HLH_glcm_MCC	Induction QT, surgery, and RT	The authors propose a radiomics model based on pretreatment with a CT radiomics signature for the detection of induction chemotherapy response in patients with locally advanced hypopharyngeal carcinoma.
Chen et al. [25] China— 2022 retrospective study	Overall survival	136	ND	58	MRI	LASSO; Original_Shape_Maximum3DDiameter, Original_Shape_Compactness, Original_Glrlma_Run-Length Non-Uniformity Normalized, Wavelet_HLLc_Glrlma_Long Run Emphasis, Wavelet_LHLd_Glcmb_Joint Entropy, Wavelet_HLHe_Glrlma_Short Run High Gray-Level Emphasis	Surgical and non-Surgical treatment	The radiomics-clinical nomogram and radiomics score might be non-invasive and reliable methods for the risk stratification in patients with hypopharyngeal squamous cell carcinoma.
Liu et al. [26] China—2022 retrospective study	Signature analysis for evaluation of response to induction chemotherapy and progression-free survival	112	103/9	61	CT	LASSO, Wavelet-LLH_glszm_ GrayLevelNonUniformityNormalized Gradient_glcm_Imc1 Wavelet-LLL_ngtdm_Busyness Wavelet-LHL_firstorder_Maximum Wavelet-LHL_firstorder_Kurtosis Wavelet-HLH_glcm_MCC	Induction chemotherapy, surgery, and RT	The authors propose that a multiparametric CT-based radiomics model could be useful for predicting treatment response and progression-free survival in patients with locally advanced hypopharyngeal carcinoma who underwent induction chemotherapy.
Nakajo et al. [27] China—2022 retrospective study	Determination of usefulness of clinical and pretreatment 18F-FDG-PET-based radiomic features for prognosis prediction in patients with hypopharyngeal cancer	100	94/6	71	PET-CT	Gray-level co-occurrence matrix entropy (GLCM_Entropy); Gray-level run-length matrix; run-length non-uniformity (GLRLM_RLNU).	Surgery, chemotherapy, CRT, RT.	The logistic regression model constructed by UICC, T and N stages and pretreatment with [18F]-FDG-PET–based radiomic features, GLCM_Entropy, and GLRLM_RLNU may be an important predictor of prognosis in patients with hypopharyngeal cancer.

**Table 2:** Assessment of Quality and Risk of Bias. OCEBM: Oxford Center for Evidence-Based Medicine; MINORS: Methodological Index for Non-Randomized Studies.

Study	OCEBM	MINORS
Chen et al. [15]	4	14
Bahig et al. [16]	4	10
Li et al. [17]	3	14
Mo et al. [18]	3	14
Hsu et al. [19]	3	12
Guo et al. [20]	4	13
Zhong et al. [21]	4	11
Fatima et al. [22]	4	11
Liu et al. [23]	3	14
Chen et al. [24]	3	14
Liu et al. [25]	3	14
Nakajo et al. [26]	3	14

## Data Availability

Data are available from the corresponding author via email.
